# Obituary

**Published:** 1906-01-15

**Authors:** 


### OBITUARY.

Dr. D. W. Clancey, of Cincinnati, died December 24th, 1905. at the age of 62 years. He had been ill for about a year. He was born in St. Albans, Vermont, December 15th,

1843. He began the practice of dentistry at Gallipolis, Ohio, where he had a large practice for several years. He graduated from the Dental Department of the University of Pennsylvania in 1870. He served in the civil war under General Shield and was wounded at Winchester, Va. He began practicing in Cincinnati in 1871 and had a very large and lucrative practice. He was an operator of fine ability, and was exceedingly careful to have his office appointments modern and tasteful. His methods of conducting his practice were always ethical, and he was noted for the attention he gave to cleanliness and sanitary methods. He was married three times and leaves a widow and three sons, one of whom is a dentist.

---

Clare G. Worboys, the eight-year old son of Dr. C. H. Worboys, of Albion, Michigan, died December 12th after a short illness. Those who know the cordial nature of Dr. Worboys will realize in a measure his great sorrow in this affliction. He will have the loving sympathy of a large number of friends.

---

Charles C. Chittenden, of Madison, Wisconsin, died Friday evening, December 15th, 1905, after an illness of several months. Dr. Chittenden was born in Livingston County, N. Y., May 10th, 1842, and was 63 years old when he died. He moved to Madison in 1855, where his father established himself as a dentist a few years before. He was educated in the high schools of Madison and the University of Wisconsin. In 1861 he entered the army and served for a little over a year as fifer in the Eleventh Wisconsin Infantry regimental band. In November 1862 the band was mustered out and he returned to Wisconsin and took up dentistry with his father. In 1866 he graduated from the Ohio Dental College. He also studied one year in the Miami Medical College at Cincinnati. He practiced with his father in Madison, until he died in 1873, and since then until his death at the same place. He was a genial man and had a host of friends. He was married May 18th, 1867, to Miss Virginia Carr Winter of New York, who died the following year. His sister Miss Kate A. Chittenden lived with him. Two other sisters are married and live in Wisconsin. He was buried at Madison December 18th. Many professional friends attended the funeral.

Dr. Chittenden has had a rather interesting career as a professional man. He has always stood for the highest type of ethics and professional attainment. He was a frequent contributor to dental literature in all branches. He was greatly interested in the educational phase of the profession and the last years of his life were devoted to placing the educational standards where he thought they should be to make the profession one of the learned callings. He loved his calling dearly and was never happier than when he was doing what he could to elevate it. As a member of the State Examining Board for his state he made a strong stand for higher registration requirements and secured a law which has been several times subjected to court rulings, because the college of his own and other states did not want to comply with its requirements. The law has always been sustained and is now accepted by all the colleges interested. Because of his work in his own state he became interested in the other state laws. Through the National Board of Examiners he has done as much as any other man to enlist the co-operation of all states in securing more uniform state legislation, and through his influence largely the National Board of Examiners has established a basis of reputability upon which it is hoped to secure in the near future more uniformity in college degree requirements than has heretofore existed. His interests have always been national as well as local. He was elected President of the National Dental Society at Asheville, and has held many offices of honor. He was a regular attendant at the National meetings and will be greatly missed at future meetings as he had lots of friends in the profession.

